# A new nomogram model for the individualized prediction of mild cognitive impairment in elderly patients with type 2 diabetes mellitus

**DOI:** 10.3389/fendo.2024.1307837

**Published:** 2024-04-09

**Authors:** Yuanyuan Jiang, Xueyan Liu, Huiying Gao, Jingzheng Yan, Yingjuan Cao

**Affiliations:** ^1^ Department of Nursing, Qilu Hospital, Shandong University, Jinan, China; ^2^ Center for Nursing Theory and Practice Innovation Research, Cheeloo College of Medicine, Shandong University, Jinan, Shandong, China; ^3^ School of Nursing and Rehabilitation, Shandong University, Jinan, Shandong, China; ^4^ Graduate School of Biomedical and Health Sciences, Hiroshima University, Hiroshima, Japan

**Keywords:** mild cognitive impairment, type 2 diabetes mellitus, elderly, model, nomogram

## Abstract

**Background:**

A high risk of developing mild cognitive impairment (MCI) is faced by elderly patients with type 2 diabetes mellitus (T2DM). In this study, independent risk factors for MCI in elderly patients with T2DM were investigated, and an individualized nomogram model was developed.

**Methods:**

In this study, clinical data of elderly patients with T2DM admitted to the endocrine ward of the hospital from November 2021 to March 2023 were collected to evaluate cognitive function using the Montreal Cognitive Assessment scale. To screen the independent risk factors for MCI in elderly patients with T2DM, a logistic multifactorial regression model was employed. In addition, a nomogram to detect MCI was developed based on the findings of logistic multifactorial regression analysis. Furthermore, the accuracy of the prediction model was evaluated using calibration and receiver operating characteristic curves. Finally, decision curve analysis was used to evaluate the clinical utility of the nomogram.

**Results:**

In this study, 306 patients were included. Among them, 186 patients were identified as having MCI. The results of multivariate logistic regression analysis demonstrated that educational level, duration of diabetes, depression, glycated hemoglobin, walking speed, and sedentary duration were independently correlated with MCI, and correlation analyses showed which influencing factors were significantly correlated with cognitive function (p <0.05). The nomogram based on these factors had an area under the curve of 0.893 (95%CI:0.856-0.930)(p <0.05), and the sensitivity and specificity were 0.785 and 0.850, respectively. An adequate fit of the nomogram in the predictive value was demonstrated by the calibration plot.

**Conclusions:**

The nomogram developed in this study exhibits high accuracy in predicting the occurrence of cognitive dysfunction in elderly patients with T2DM, thereby offering a clinical basis for detecting MCI in patients with T2DM.

## Introduction

Diabetes as a chronic metabolic disease is considered a major public health challenge. Currently, diabetes affects more than 400 million people worldwide, and it is projected that the global diabetes prevalence will increase from 9.3% (463 million) in 2019 to 10.2% (578 million) in 2030 and 10.9% (700 million) in 2045 (1). Type 2 diabetes mellitus (T2DM), characterized by insulin resistance, is a form of diabetes that accounts for 90% of all individuals with diabetes (2). Furthermore, studies have demonstrated that China ranks the second globally in annual health expenditure on diabetes (3). It is also the country with the highest number of elderly patients with diabetes, with a diabetes prevalence of 28.8% among people aged 60–69 years and 31.8% among those aged 70 years and older (4). T2DM is prone to several complications, such as cardiovascular disease, peripheral neuropathy, and cognitive impairment (5).

Meanwhile, a growing body of clinical and epidemiologic data indicates that patients with T2DM have a higher incidence of cognitive impairment than non-diabetic patients (6). Patients with diabetes and prediabetes face a 1.25- to 1.91-fold increased risk of developing cognitive impairment (4), such as mild cognitive impairment (MCI) and dementia. Studies have also demonstrated that the prevalence of MCI in patients with T2DM is 45% (7).

MCI is a transitional stage between cognitive normalcy and dementia with bi-directional transformation, MCI is a progressive loss of memory or other cognitive function that does not affect daily life functioning and does not meet the diagnostic criteria for dementia (8), and as a common central nervous system complication in elderly patients with T2DM, the two interact with each other. The general MCI criteria include the following: (i) clinical concern raised by the patient or an informant, or observations made by the clinician, (ii) cognitive impairment in one or more cognitive domains preferably relative to appropriate normative data for that individual, (iii) preservation of functional independence and (iv) no dementia (9). Diabetes is an independent risk factor for the progression of dementia in patients with MCI (10). Factors such as impaired insulin signaling pathway, inflammation, accumulation of advanced glycosylation end-products, and oxidative stress (11) can result in a 2.01- to 2.84-fold increase in the risk of developing MCI (12–14) and accelerate the transition of MCI to dementia (14, 15). On the other hand, decreased self-management and increased dependence on care can be experienced by patients with MCI due to impaired attention, executive function, memory, and decision-making ability. This not only complicates the management of diabetes but also has been linked to an increased mortality rate in patients with MCI (16, 17). Thus, MCI, as a prodromal stage of dementia, has become the primary focus for early prevention and treatment of dementia in elderly patients with T2DM. Early identification of patients at high risk of cognitive impairment is crucial for enhancing their clinical prognosis.

Current research is concentrating on the risk factors of MCI in elderly patients with T2DM, and clinical practice still faces the challenge of determining how to use quantitative indicators to achieve individualized prediction. The nomogram model can quantify, visualize, and graphically present the findings of multifactorial logistic regression, enabling individualized prediction of the risk of a specific clinical event. Individualized prediction has successfully found applications in assessing the occurrence, prognosis, and recurrence of various clinical events (18, 19). To date, no nomogram model has been developed for predicting the risk of MCI in elderly patients with T2DM. Thus, this study aims to examine the independent risk factors associated with MCI in elderly patients with diabetes and to develop a simple and practical nomogram for predicting cognitive impairment in elderly patients with T2DM, with the aim of early identification of patients at high risk of cognitive impairment and offering a reference for enhancing disease management and clinical interventions.

## Methods

### Patients and selection criteria

This study enrolled elderly patients with T2DM who were hospitalized in the Department of Endocrinology at Qilu Hospital of Shandong University from November 2021 to March 2023, resulting in 306 patients. Inclusion criteria: (1) Age ≥60 years old; (2) met the T2DM diagnostic criteria outlined in the Chinese Guidelines for the Prevention and Treatment of Type 2 Diabetes (2020 Edition) (20); (3) voluntary participation in the study. The exclusion criteria were: (1) Patients with severe visual and auditory impairment, mental abnormality, or unclear expression; (2) patients with malignant tumors, severe liver and renal impairment, or other serious complications; (3) patients with alcohol dependence or a history of psychoactive drug abuse; (4) patients with neurological lesions (e.g., stroke, brain tumors, and Parkinson’s disease) that can result in cognitive dysfunction; and (6) patients with diagnosed dementia.

### Sample size calculation

According to Moving beyond the 10 events per variable (10 EPV) rule of thumb (21), the number of independent variables included in this study was 16, with a minimum sample size of 160 patients.

### Study variables

Variables examined in this study included age, sex, marital status, educational level, location of residence, living situation, current smoking, current alcohol consumption, body mass index, number of chronic diseases, duration of diabetes (years), hypoglycemia (within 3 months), depression, glycated hemoglobin (HbA1c), walking speed (measured over 4 m), and sedentary duration.

### Cognitive function assessment

The Montreal Cognitive Assessment (MoCA) Scale (22) was used to evaluate eight cognitive domains, including abstract thinking, attention and concentration, executive function, language, memory, visual structure skills, computation, and orientation. Scores ranging from 0 to 30 were used, with better cognitive functioning indicated by higher scores. A score of ≥26 on the MoCA was considered normal, and a total score of <26 indicates MCI. If the number of years of education was less than 12 years, one point was added to the results to correct for educational bias.

The Mini-Mental State Examination (MMSE) scale is commonly used in studies and clinical assessment of general cognitive function, with a maximum score of 30 points. It contains 5 subscales, Orientation, Registration, Attention and Calculation, Recall, Language, and Visual Construction, with higher scores indicating better cognitive function (23). Its total score ranged from 0 to 30, with total scores ≤19 for illiterate, ≤22 for individuals with elementary school education, and ≤26 for individuals with junior high school or higher education categorized as MCI (24).

### Depressive symptom

The depression status of elderly patients was assessed using the 5-item Geriatric Depression Scale (GDS-5) (25). The scale is a simplified version of GDS-15, assigning a score of 1 to each entry, resulting in a total score ranging from 0 to 5, with higher scores indicating more severe depression and a score of ≥2 indicating the presence of depressive symptoms. GDS-5 is more concise and convenient to use and has good reliability and validity (26). Therefore, it has been extensively employed in the screening of depressive symptoms in the elderly population.

### Data collection

Uniformly trained enumerators conducted face-to-face interviews using a paper version of the questionnaire to retrospectively collect relevant data. All questionnaires were distributed, completed, and returned on-site, and two individuals entered and cross-checked the data.

### Statistical analysis

SPSS 25.0 was used to perform statistical analysis of the data. Categorical variables were expressed as frequencies (percentages) and continuous variables as mean ± standard deviation or median (quartiles). The Mann-Whitney U test (for non-normally distributed variables) or the t-test (for normally distributed variables) was used to compare continuous variables, and the chi-square test or Fisher’s exact test was used to compare categorical variables. Clinical factors with statistically significant (*p* < 0.05) differences in the results of the one-way logistic regression analyses were subsequently included in the multifactorial logistic regression analysis, and associations were assessed using Spearman’s rank correlation coefficient (*p* < 0.05).

The nomogram of the prediction model was constructed using the R software package “rms” based on the screened independent risk factors. The bootstrap method with 1000 replicates was used to perform internal validation of the nomogram model in the original sample. The receiver operator characteristic (ROC) curve was used to evaluate the predictive ability of the model, and the area under the curve (AUC) was computed to evaluate model performance. The closer the AUC is to 1 (AUC value > 0.75), the better the discriminative validity of the model. The calibration was constructed to examine the agreement between the predicted probabilities with the observed outcome, and a calibration curve was constructed and a Hosmer-Lemeshow test was performed to evaluate the calibration of the nomogram. In addition, a decision curve analysis (DCA) of the model to assess the clinical validity of the model. All statistical tests were two-tailed, and p-values <.05 were considered statistically significant.

## Results

### Characteristics of the participants

After rigorously screening the study population based on the inclusion and exclusion criteria, 306 elderly patients with T2DM were enrolled. Based on the MOCA score, 186 of the 306 patients were categorized as MCI, while 120 were classified as normal cognitive. The mean age of the patients (including 175 males and 131 females) was 66.19 ± 1.92 (years). **Table 1** summarizes the characteristics of the study population. The differences between the study population in terms of gender, literacy level, alcohol consumption status, depressive symptoms, duration of diabetes, sedentary time, step speed, and HbA1c, and the difference between the MCI normocognitive groups were significant (*p* < 0.05).

**Table 1:** Characteristics of the study population by mild cognitive impairment (MCI) status.

Factors	Description	MCI status		Test statisic	*p* value
		Normal cognition group (n =120)	MCI group (n =186 )		
Age (years)		65.67±5.88	66.53±5.93	-1.251	.212
Sex				11.567	.001
	male	83 (69.2%)	92 (49.5%)		
	Female	37 (30.8%)	94 (50.5%)		
Marital status				1.844	.174
	Married	111 (92.5%)	163 (87.6%)		
	Divorced/ Widowed	9 (7.5%)	23 (12.4%)		
Educational level				24.934	<.001
	Elementary school or below	10 (8.3%)	43 (23.1%)		
	Middle school	17 (14.2%)	51 (27.4%)		
	High school	50 (41.7%)	54 (29.0%)		
	College or above	43 (35.8%)	38 (20.4%)		
Location of residence				0.556	.456
	Urban area	102 (85.0%)	152 (81.7%)		
	Rural area	18 (15.0%)	34 (18.3%)		
Living situation				0.773	.379
	Living alone	11 (9.2%)	12 (6.5%)		
	Not living alone	109 (90.8%)	174 (93.5%)		
Current smoking				2.063	.151
	No	73 (60.8%)	128 (68.8%)		
	Yes	47 (39.2%)	58 (31.2%)		
Current alcohol consumption				9.293	.002
	No	59 (49.2%)	124 (66.7%)		
	Yes	61 (50.8%)	62 (33.3%)		
BMI				7.129	.068
	≤18.4	1 (0.8%)	5 (2.7%)		
	18.5-24.9	55 (45.8%)	109 (58.6%)		
	25-29.9	53 (44.2%)	58 (31.2%)		
	≥30	11 (9.2%)	14 (7.5%)		
Number of chronic diseases				5.470	0.140
	0	18 (15.0%)	13 (7.0%)		
	1	25 (20.8%)	46 (24.7%)		
	2	29 (24.2%)	44 (23.7%)		
	≥3	48 (40.0%)	82 (44.6%)		
Hypoglycemia (within 3 months)				1.217	.270
	No	81 (67.5%)	114 (61.3%)		
	Yes	39 (32.5%)	72 (38.7%)		
Depression				16.209	<.001
	No	99 (82.5%)	113 (60.8%)		
	Yes	21 (17.5%)	73 (39.2%)		
HbA1c		7.38±1.32	8.89±1.67	-8.294	<.001
Walking speed		1.03±0.24	1.19±0.35	-4.190	<.001
Sedentary duration		4.00 (2.125, 6.00)	9.00 (6.00, 12.00)	8.967	<.001
Duration of diabetes (years)		10.00 (5.00, 16.00)	14.00 (10.00, 20.00)	2.840	<.001

BMI, body mass index; HbA1c, glycated haemoglobin.

### Logistic univariate and multivariate regression analysis results

**Table 2** presents the results. Variables that showed statistical significance in the univariate analysis were included in the multifactorial logistic regression analysis, which demonstrated that the development of MCI in elderly patients with T2DM was influenced by educational level, duration of diabetes, depression, HbA1c, walking speed, and sedentary duration. Risk factors for the development of cognitive dysfunction in patients included the presence of depressive symptoms, longer duration of diabetes, extended sedentary time, slower walking speed, and high levels of HbA1c (*p* < 0. 05), whereas higher education was identified as a protective factor.

**Table 2:** Logistic univariate and multivariate regression analysis of MCI in patients.

Factors	Description	Univariate logistic regression		Multivariate logistic regression	
		OR (95% CI)	*P* value	OR (95% CI)	*P* value
Sex	male	Reference		Reference	
	Female	0.436 (0.269-0.707)	0.001	0.598 (0.253-1.413)	.241
Educational level	Elementary school or below	Reference		Reference	
	Middle school	4.866 (2.154-10.991)	<.001	2.669 (0.899-7.024)	.077
	High school	3.395 (1.684-6.845)	.001	2.755 (1.036-7.326)	.042
	College or above	1.222 (0.683-2.186)	.499	0.829 (0.376-1.829)	.643
Drinking	No	Reference		1.694 (0.741-3.871)	.002
	Yes	2.068 (1.292-3.309)	.002		
Depression	No	Reference		Reference	
	Yes	0.328 (0.188-0.572)	<.001	0.416 (0.189-0.915)	.029
Walking speed		6.020 (2.446-14.813)	<.001	8.381 (2.648-26.528)	<.001
HbA1c		2.135 (1.724-2.642)	<.001	1.929 (1.514-2.458)	<.001
Sedentary duration		1.333 (1.234-1.440)	<.001	1.339 (1.220-1.469)	<.001
Duration of diabetes (years)		1.078 (1.042-1.115)	<.001	1.061 (1.015-1.109)	.009

HbA1c, glycated haemoglobin.

### Correlation analysis results

In this study, MoCA was significantly correlated with education (*r*=0.272, *p*<0.05), duration of diabetes (*r*=-0.279, *p*<0.05), depression (*r*=-0.230, *p*<0.05), HbA1c (*r*=-0.490, *p*<0.05), walking speed (*r*=-0.247, *p*<0.05), sedentary time (*r*=-0.484, *p*<0.05) (**Table 3**). MMSE was significantly correlated with education (r=0.369, p<0.05), duration of diabetes (r=-0.126, p<0.05), depression (r=-0.160, p<0.05), HbA1c (r=-0.281, p<0.05), walking speed (r=-0.226, p<0.05), sedentary time (r=-0.159, p<0.05) (**Table 4**).

**Table 3:** Correlation between cognitive level and influence factors.

Variable	*r*	*p* value
Educational level	0.272	<.05
Duration of diabetes (years)	-0.279	<.05
Depression	-0.230	<.05
HbA1c	-0.490	<.05
Walking speed	-0.247	<.05
Sedentary duration	-0.484	<.05

HbA1c, glycated haemoglobin.

**Table 4:** Correlation between MMSE and influence factors.

Variable	*r*	*p* value
Educational level	0.369	<.05
Duration of diabetes (years)	-0.126	<.05
Depression	-0.160	<.05
HbA1c	-0.281	<.05
Walking speed	-0.226	<.05
Sedentary duration	-0.159	<.05

HbA1c, glycated haemoglobin.

### Construction of the nomogram

**Figure 1:**
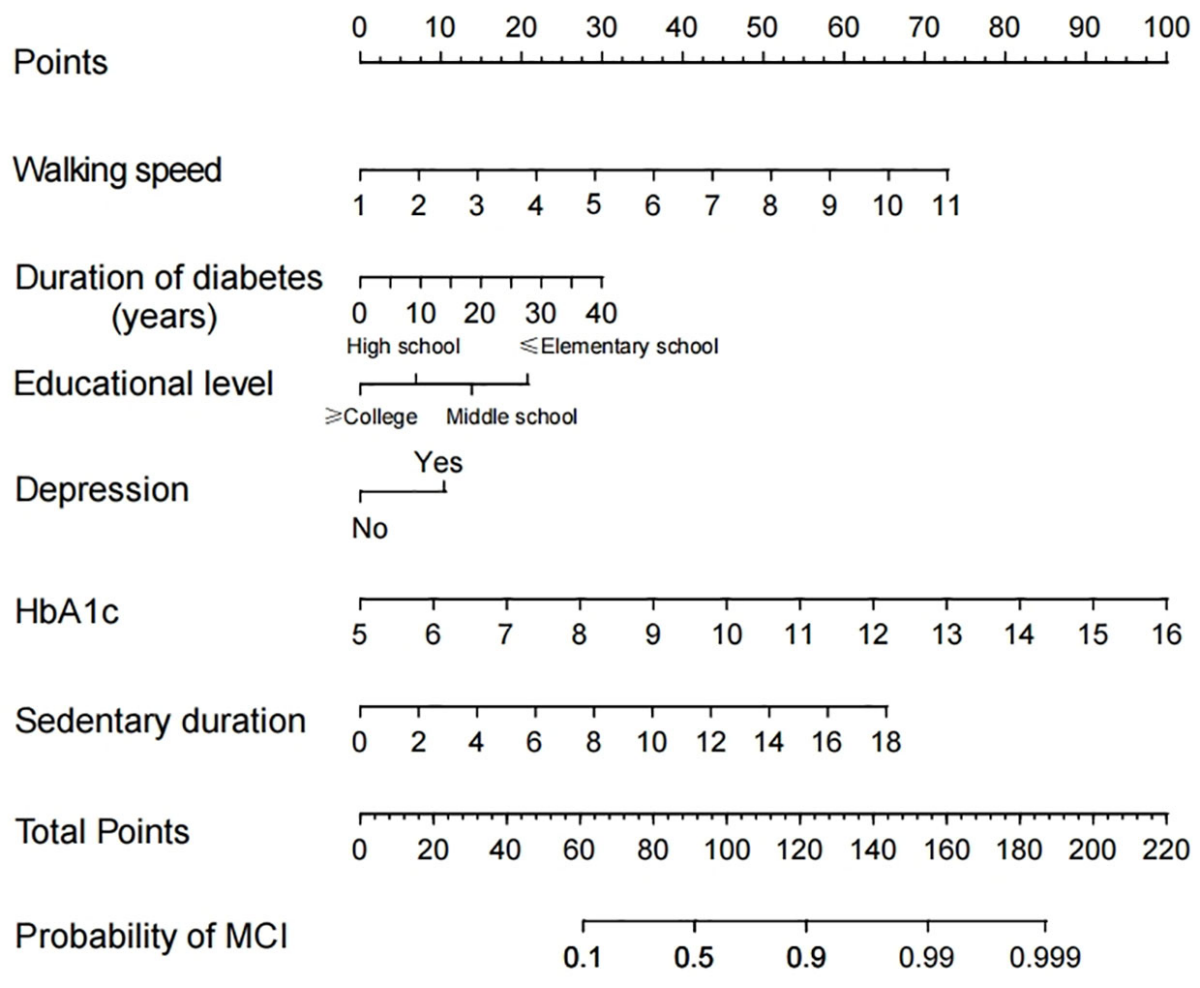
Nomogram to detect mild cognitive impairment (MCI) in elderly Patients with Type 2 Diabetes Mellitus (T2DM).

In this study, a line graph model was developed for predicting the risk of MCI in elderly patients with T2DM based on six independent risk factors for MCI: Literacy, disease duration, depressive symptoms, HbA1c, walking speed, and sedentary time (**Figure 1**).

### Internal validation of the nomogram and model performance

**Figure 2:**
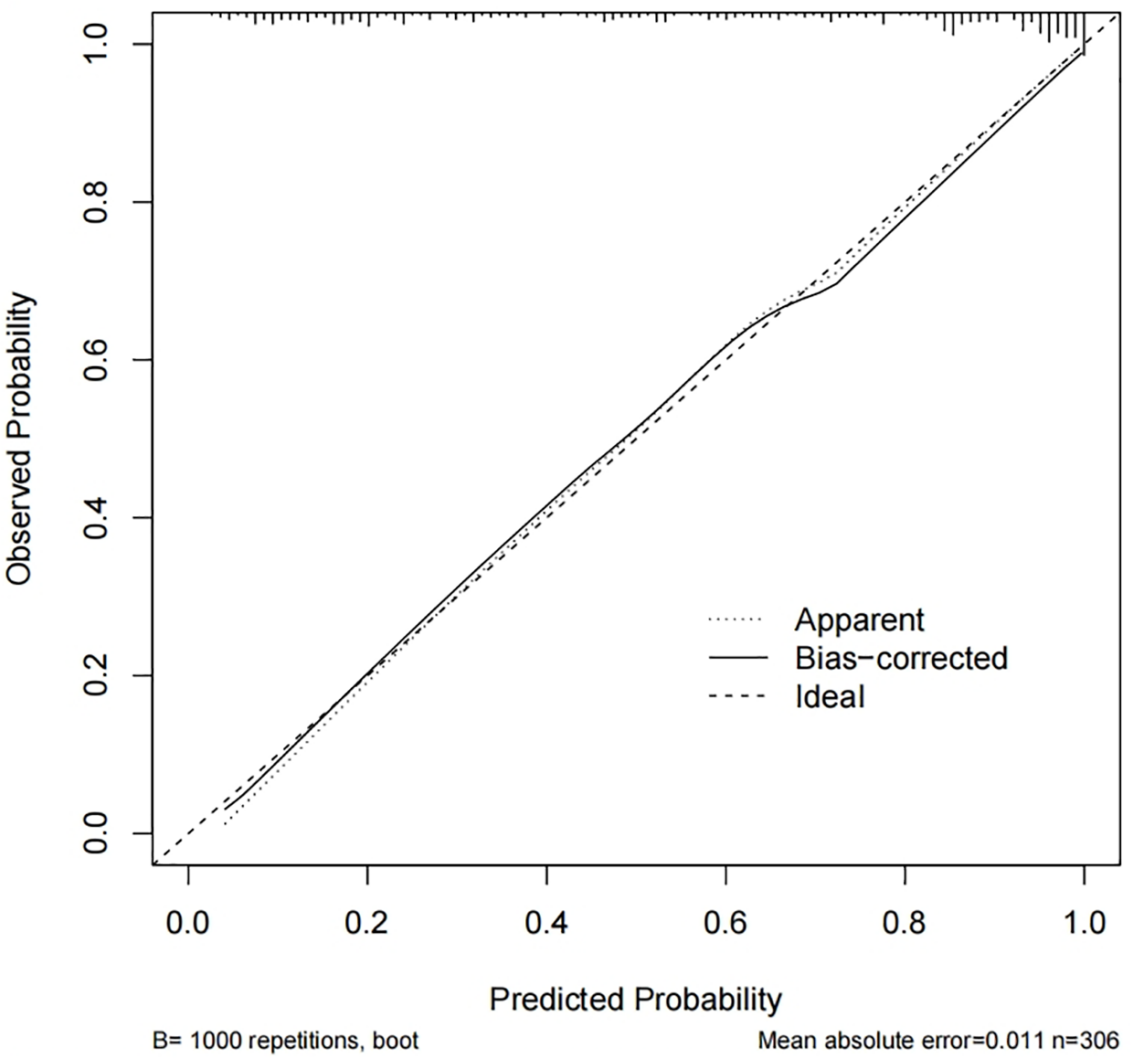
Calibration curve for internal validation of the nomogram.

**Figure 3:**
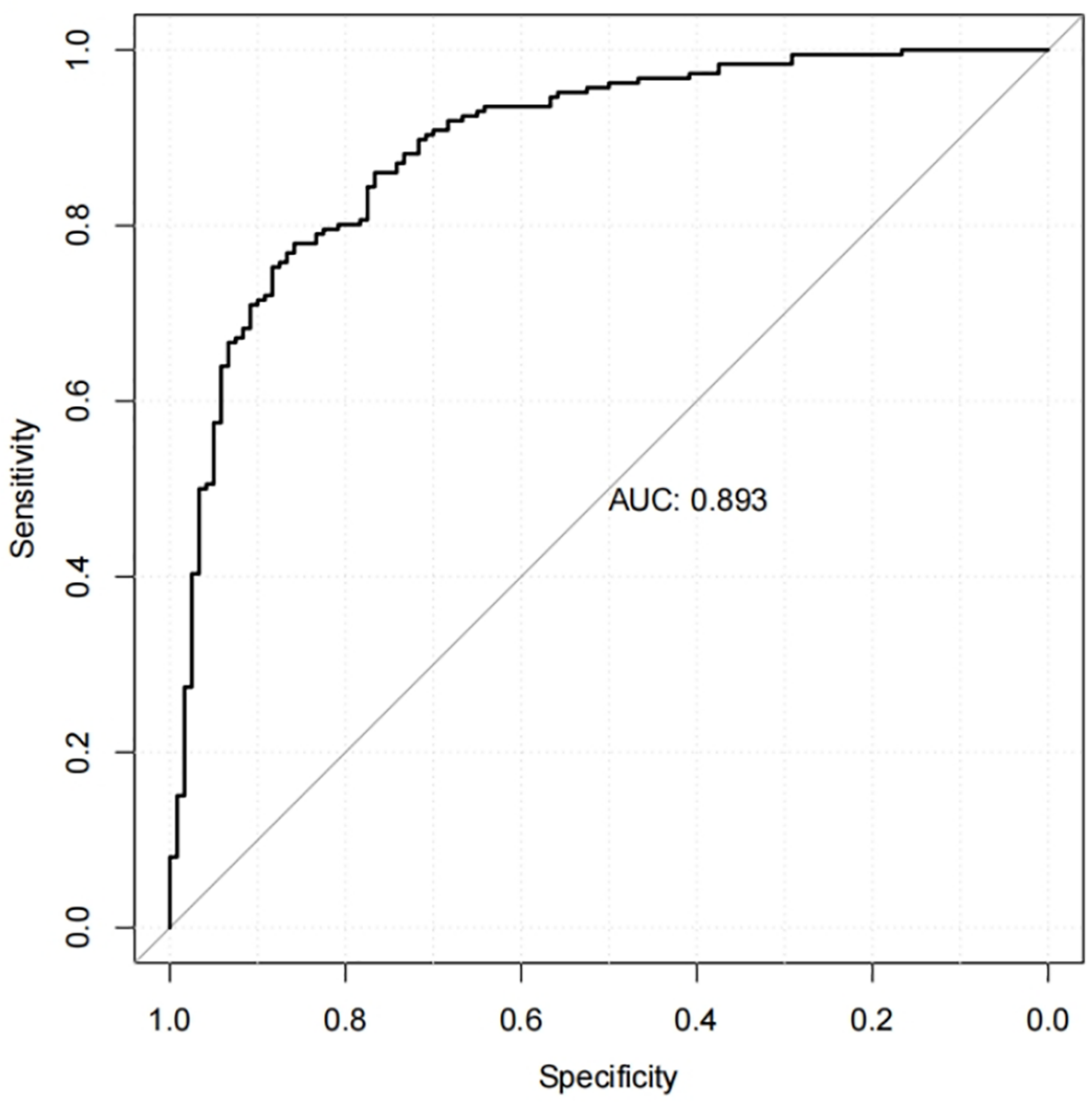
The receiver operating characteristic curve of the nomogram.

Calibration curves were used to assess the accuracy of the predicted MCI risk column plot model (**Figure 2**). The X-axis represents the predicted MCI risk, and the Y-axis represents the risk of actual MCI diagnosis. An ideal model with perfect predictive power is represented by the diagonal dashed line, and the solid line represents the column line graph model in this study. In a well-calibrated model, the detection should correspond to the 45th diagonal. A closer fit to the diagonal dashed line indicated better prediction, and this study demonstrates good agreement between the constructed model and real observations. **Figure 3** shows the ROC curve with an AUC value of 0.893 (95%*CI*:0.856-0.930) (*p*<0.05), and the sensitivity and specificity were 0.785 and 0.850, respectively. The Hosmer-Lemeshow test results indicated the absence of statistical significance (*χ* ^2 = ^7.337, *p=*0.501) (*p*>0.05), indicating that the nomogram provided a good fit to the data.

### Clinical utility of the nomogram

**Figure 4:**
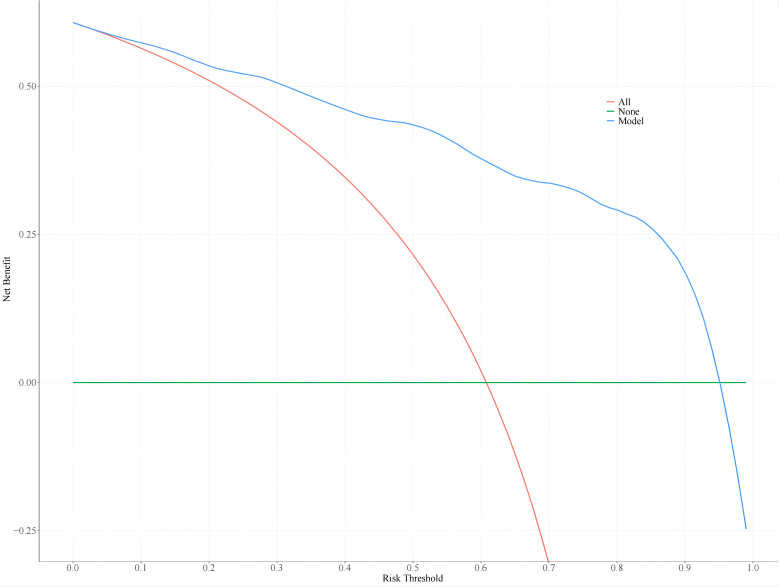
The decision curve analysis of the nomogram.

Nomogram threshold probabilities ranged from 5.0% to 95.0% by DCA. Elderly patients with T2DM had a higher net benefit than the other two extreme curves, indicating the clinical validity of the model. The clinical utility of the nomogram is shown in **Figure 4**.

## Discussion

Chronic damage and dysfunction of blood vessels, nerves, brain, and other tissues and organs are often caused by insufficient insulin secretion or insulin resistance in patients with T2DM, leading to impaired cognitive function (7). Furthermore, T2DM in elderly individuals accelerates the onset of MCI or increases the risk of dementia development at a higher rate (27). In this study, the detection rate of MCI in elderly patients with T2DM was 51.67%, surpassing the global combined prevalence of MCI in elderly patients with T2DM, which is 45% (7). Currently, there is a lack of effective treatment for cognitive impairment, and in severe cases, progression to dementia may occur. Consequently, great clinical importance is attached to the early-stage identification of factors influencing the occurrence of MCI in patients with T2DM and the search for indicators with predictive and diagnostic value for MCI.

In this study, a nomogram-based model for predicting the risk of MCI in elderly patients with T2DM was developed and validated. This model combines educational level, disease duration, depressive symptoms, walking speed, sedentary time, and HbA1c and exhibits good discriminative ability, accuracy, and clinical applicability. This provides clinicians with a potentially simple and easy-to-use tool for predicting the risk of MCI in elderly patients with T2DM. Furthermore, it serves as a research basis for conducting cognitive function follow-up, initiating cognitive function training as early as possible, and slowing deterioration.

The findings of this study indicate a significant association between educational level and T2DM-MCI. Some studies have validated the close relationship between the severity of cognitive impairment in patients with T2DM and their educational level (28). This association may be attributed to the fact that individuals with higher levels of education, particularly knowledge workers, tend to exhibit higher synaptic densities in the cortex, resulting in increased brain storage. Engaging the brain in various cognitive tasks allows brain cells to receive more stimuli, thereby promoting the maintenance of cognitive functions in the brain (29–31). Highly educated individuals often possess sufficient cognitive reserve, which contributes to a slower rate of cognitive decline. Older adults may delay cognitive decline by continuing to improve their intellectual abilities throughout their lifespan (32). Thus, older adults with T2DM are encouraged to change their lifestyles, learn new things, embrace new experiences, receive more cognitive stimulation, and actively engage their brains throughout their lifespan to potentially prevent cognitive decline.

The findings of this study indicated that the duration of diabetes is a significant risk factor for the development of MCI in patients with T2DM, and a positive correlation between the duration of diabetes and cognitive decline in patients with T2DM was identified (33). Prospective studies have also concluded that individuals with a T2DM duration exceeding 5 years have a 1.59 times greater risk of developing cognitive impairment than others (13). Prolonged disease duration in patients with T2DM is accompanied by memory loss and a substantial decline in visual and spatial judgment (28). The extended duration of T2DM, macrovascular and microvascular disease, and oxidative stress injury may increase neuronal damage. Furthermore, long-term insulin resistance and poor glycemic control lead to an increase in late-onset glycosylation end products (34), which contribute to an increase in T2DM complications and cognitive dysfunction.

Previous studies have consistently demonstrated a strong association between depressive symptoms and cognitive decline in patients with T2DM (35, 36). A recent systematic review and meta-analysis revealed that depression not only increases the risk of cognitive decline and dementia but also accelerates the conversion of MCI to dementia (37), which may be explained by the influence of depressive symptoms on increasing the amyloid burden in the cerebral cortex and reducing synaptic plasticity (38, 39). Consistent with previous results, this study also identified a correlation between depressive symptoms and T2DM-MCI in older adults, emphasizing the importance of regular screening for depressive symptoms and timely intervention when necessary.

HbA1c is a crucial indicator of glycemic control in patients with T2DM, and its higher levels indicate that oxidative stress, neuronal damage, and vascular damage in the body are more severe, potentially leading to more severe cognitive dysfunction (40). In an Advanced Chronic Kidney Disease Study, interventions to lower HbA1c levels did not affect cognitive decline (41). However, a Diabetes Education and Telemedicine Study (IDEATel) demonstrated that cognitive decline was observed with interventions at HbA1c <7.0% (42). In this study, the higher the HbA1c level, the more likely the patient was to develop MCI, indicating a bidirectional relationship between cognitive impairment and diabetes management. Impaired cognitive function can result in a decrease in the ability of diabetic patients to self-manage their diabetes, leading to unstable glycemic control and a higher likelihood of complications such as hypoglycemia, which can exacerbate cognitive impairment (43). The advantages of glycemic control in older adults with T2DM are indisputable, and future studies should aim to determine the optimal level of glycemic control for older adults. Furthermore, future studies should investigate the long-term effects of maintaining optimal glycemic control on cognitive decline in patients. Some studies have shown that homocysteine levels may be an influential factor in cardiovascular disease (44), so homocysteine levels may affect cognitive function in elderly patients with T2DM, we will also consider the effect of homocysteine and other biochemical indexes on cognitive levels in our next study.

Studies have demonstrated that grip strength and walking speed are positively related to overall cognitive function (45), lower daily walking speed is correlated with more severe cognitive impairment (46), and the effect of walking speed on cognitive function may be more pronounced in the later stages of cognitive impairment (47, 48), These findings are consistent with the results of this study, which employed walking speed as a predictor of MCI in patients. Consequently, clinical care providers should consider improving cognitive and physical function when devising relevant interventions. These findings indicate that clinical care providers should consider the influences of cognitive and physical function when developing relevant interventions and adopt interventions that combine cognition and exercise, such as TaiChi exercises that encompass balance and aerobic function. Furthermore, special attention should be directed toward the care of individuals who have sustained fall injuries due to a decrease in walking speed.

The poor lifestyle of sedentary older individuals with T2DM exposes them to poor glycemic control. Unstable blood glucose levels (including higher than average blood glucose levels and increased blood glucose variability) have been significantly associated with cognitive impairment (49, 50). Furthermore, an increased likelihood of cognitive impairment has been associated with spending a substantial amount of time sitting, irrespective of whether the overall activity level is high or low (51). Fritschi’s study discovered that the longer the sedentary time, the longer the hyperglycemia, leading to an increase in anaerobic metabolism and subsequent damage to hypoxic brain tissue, which may eventually result in cognitive impairment (52). Similarly, this study also confirmed a significant association between sedentary duration and the development of MCI in elderly patients with T2DM, indicating that a longer sedentary duration corresponds to a higher risk of MCI. Thus, community health workers should prioritize monitoring sedentary behavior and integrating it into the disease management program for elderly patients with T2DM. Efforts should be made to reduce the duration of sedentary behavior by strengthening health education and implementing strategies to interrupt sedentary behavior, which in turn reduces the risk of cognitive impairment in patients.

In conclusion, a nomogram of the prediction model based on six independent risk factors in elderly patients with T2DM was developed in this study. Calibration and ROC curves were used to evaluate the accuracy of the model. The findings indicated that the model exhibited good accuracy, discriminative ability, and clinical validity. Furthermore, the results demonstrate that early evaluation of cognitive function in elderly patients with T2DM and early clinical interventions targeting relevant independent risk factors are particularly important in the evaluation and management of patients’ conditions. This model can serve as a guide for improved clinical decision-making and individualized intervention management.

## Conclusions

In this study, a risk model for MCI in elderly patients with T2DM was developed, incorporating educational level, duration of diabetes, depression, HbA1c, walking speed, and sedentary duration as key factors. The application of a nomogram to evaluate the risk of MCI development in elderly patients with T2DM is a novel concept that may produce reliable and optimal models. These models can offer valuable guidance for enhancing clinical decision-making and implementing individualized intervention management strategies. However, this study has some limitations. First, the nomogram model developed in this study, which used clinical data from one hospital, underwent only internal validation and lacked validation using external clinical data from other clinical sources. Second, the sample size of the clinical data in this study is limited and requires expansion to enable further validation. Finally, the clinical and biochemical characteristics of the included samples were not adequately considered in this study and we will perform additional analyses in future studies.

## Data availability statement

The raw data supporting the conclusions of this article will be made available by the authors, without undue reservation.

## Ethics statement

The studies involving humans were approved by Qilu Hospital of Shandong University. The studies were conducted in accordance with the local legislation and institutional requirements. The participants provided their written informed consent to participate in this study.

## Author contributions

YJ: Writing – review & editing, Writing – original draft. XL: Writing – review & editing, Software, Methodology. HG: Writing – review & editing, Investigation, Data curation. JY: Writing – review & editing, Investigation. YC: Writing – review & editing, Supervision.

## References

[1] Sun H Saeedi P Karuranga S Pinkepank M Ogurtsova K Duncan BB. IDF Diabetes Atlas: Global, regional and country-level diabetes prevalence estimates for 2021 and projections for 2045. Diabetes Res Clin Pract. (2022) 183:109119. doi: 10.1016/j.diabres.2021.109119 34879977 PMC11057359

[2] Zheng Y Ley SH Hu FB. Global aetiology and epidemiology of type 2 diabetes mellitus and its complications. Nat Rev Endocrinol. (2018) 14:88–98. doi: 10.1038/nrendo.2017.151 29219149

[3] Williams R Karuranga S Malanda B Saeedi P Basit A Besançon S. Global and regional estimates and projections of diabetes-related health expenditure: Results from the International Diabetes Federation Diabetes Atlas, 9th edition. Diabetes Res Clin Pract. (2020) 162:108072. doi: 10.1016/j.diabres.2020.108072 32061820

[4] Sinclair A Saeedi P Kaundal A Karuranga S Malanda B Williams R. Diabetes and global ageing among 65-99-year-old adults: Findings from the International Diabetes Federation Diabetes Atlas, 9(th) edition. Diabetes Res Clin Pract. (2020) 162:108078. doi: 10.1016/j.diabres.2020.108078 32068097

[5] Nanda M Sharma R Mubarik S Aashima A Zhang K. Type-2 diabetes mellitus (T2DM): spatial-temporal patterns of incidence, mortality and attributable risk factors from 1990 to 2019 among 21 world regions. Endocrine. (2022) 77:444–54. doi: 10.1007/s12020-022-03125-5 35841511

[6] Degen C Toro P Schönknecht P Sattler C Schröder J. Diabetes mellitus Type II and cognitive capacity in healthy aging, mild cognitive impairment and Alzheimer's disease. Psychiatry Res. (2016) 240:42–6. doi: 10.1016/j.psychres.2016.04.009 27082868

[7] You Y Liu Z Chen Y Xu Y Qin J Guo S. The prevalence of mild cognitive impairment in type 2 diabetes mellitus patients: a systematic review and meta-analysis. Acta diabetologica. (2021) 58:671–85. doi: 10.1007/s00592-020-01648-9 33417039

[8] Langa KM Levine DA. The diagnosis and management of mild cognitive impairment: a clinical review. Jama. (2014) 312:2551–61. doi: 10.1001/jama.2014.13806 PMC4269302 25514304

[9] Petersen RC Caracciolo B Brayne C Gauthier S Jelic V Fratiglioni L. Mild cognitive impairment: a concept in evolution. J Internal Med. (2014) 275:214–28. doi: 10.1111/joim.12190 PMC3967548 24605806

[10] Ciudin A Espinosa A Simó-Servat O Ruiz A Alegret M Hernández C. Type 2 diabetes is an independent risk factor for dementia conversion in patients with mild cognitive impairment. J Diabetes its complications. (2017) 31:1272–4. doi: 10.1016/j.jdiacomp.2017.04.018 28545893

[11] Srikanth V Sinclair AJ Hill-Briggs F Moran C Biessels GJ. Type 2 diabetes and cognitive dysfunction-towards effective management of both comorbidities. Lancet Diabetes Endocrinol. (2020) 8:535–45. doi: 10.1016/s2213-8587(20)30118-2 32445740

[12] Ng TP Feng L Nyunt MS Feng L Gao Q Lim ML. Metabolic syndrome and the risk of mild cognitive impairment and progression to dementia: follow-up of the Singapore longitudinal ageing study cohort. JAMA Neurol. (2016) 73:456–63. doi: 10.1001/jamaneurol.2015.4899 26926205

[13] Rawlings AM Sharrett AR Albert MS Coresh J Windham BG Power MC. The association of late-life diabetes status and hyperglycemia with incident mild cognitive impairment and dementia: the ARIC study. Diabetes Care. (2019) 42:1248–54. doi: 10.2337/dc19-0120 PMC6609963 31221696

[14] Dove A Shang Y Xu W Grande G Laukka EJ Fratiglioni L. The impact of diabetes on cognitive impairment and its progression to dementia. Alzheimer's dementia J Alzheimer's Assoc. (2021) 17:1769–78. doi: 10.1002/alz.12482 34636485

[15] Ma F Wu T Miao R Xiao YY Zhang W Huang G. Conversion of mild cognitive impairment to dementia among subjects with diabetes: a population-based study of incidence and risk factors with five years of follow-up. J Alzheimer's Dis JAD. (2015) 43:1441–9. doi: 10.3233/jad-141566 25159674

[16] Martinez M Dawson AZ Lu K Walker RJ Egede LE. Effect of cognitive impairment on risk of death in Hispanic/Latino adults over the age of 50 residing in the United States with and without diabetes: Data from the Health and Retirement Study 1995-2014. Alzheimer's dementia J Alzheimer's Assoc. (2022) 18:1616–24. doi: 10.1002/alz.12521 PMC9170835 34873809

[17] Świątoniowska-Lonc N Polański J Tański W Jankowska-Polańska B. Impact of cognitive impairment on adherence to treatment and self-care in patients with type 2 diabetes mellitus. Diabetes Metab syndrome Obes Targets Ther. (2021) 14:193–203. doi: 10.2147/dmso.S284468 PMC7815078 33488107

[18] Wang H Ou Y Fan T Zhao J Kang M Dong R. Development and internal validation of a nomogram to predict mortality during the ICU stay of thoracic fracture patients without neurological compromise: an analysis of the MIMIC-III clinical database. Front Public Health. (2021) 9:818439. doi: 10.3389/fpubh.2021.818439 35004604 PMC8727460

[19] Liu Z Li C. A predictive model for the risk of cognitive impairment in patients with gallstones. BioMed Res Int. (2021) 2021:3792407. doi: 10.1155/2021/3792407 34337006 PMC8313337

[20] Chinese Elderly Type 2 Diabetes Prevention and Treatment of Clinical Guidelines Writing Group; Geriatric Endocrinology and Metabolism Branch of Chinese Geriatric Society; Geriatric Endocrinology and Metabolism Branch of Chinese Geriatric Health Care Society; Geriatric Professional Committee of Beijing Medical Award Foundation; National Clinical Medical Research Center for Geriatric Diseases (PLA General Hospital). [Clinical guidelines for prevention and treatment of type 2 diabetes mellitus in the elderly in China (2022 edition)]. Zhonghua Nei Ke Za Zhi. (2022) 61(1):12–50. Chinese. doi: 10.3760/cma.j.cn112138-20211027-00751. 34979769

[21] Riley RD Ensor J Snell KIE Harrell FE Jr. Martin GP Reitsma JB. Calculating the sample size required for developing a clinical prediction model. BMJ. (2020) 368:m441. doi: 10.1136/bmj.m441 32188600

[22] Nasreddine ZS Phillips NA Bédirian V Charbonneau S Whitehead V Collin I. The Montreal Cognitive Assessment, MoCA: a brief screening tool for mild cognitive impairment. J Am Geriatrics Soc. (2005) 53:695–9. doi: 10.1111/j.1532-5415.2005.53221.x 15817019

[23] Folstein MF Folstein SE McHugh PR. "Mini-mental state". A practical method for grading the cognitive state of patients for the clinician. J Psychiatr Res. (1975) 12:189–98. doi: 10.1016/0022-3956(75)90026-6 1202204

[24] Li H Jia J Yang Z. Mini-mental state examination in elderly Chinese: A population-based normative study. J Alzheimer's Dis JAD. (2016) 53:487–96. doi: 10.3233/jad-160119 27163822

[25] Hoyl MT Alessi CA Harker JO Josephson KR Pietruszka FM Koelfgen M. Development and testing of a five-item version of the Geriatric Depression Scale. J Am Geriatrics Soc. (1999) 47:873–8. doi: 10.1111/j.1532-5415.1999.tb03848.x 10404935

[26] Rinaldi P Mecocci P Benedetti C Ercolani S Bregnocchi M Menculini G. Validation of the five-item geriatric depression scale in elderly subjects in three different settings. J Am Geriatrics Soc. (2003) 51:694–8. doi: 10.1034/j.1600-0579.2003.00216.x 12752847

[27] Kim HG. Cognitive dysfunctions in individuals with diabetes mellitus. Yeungnam Univ J Med. (2019) 36:183–91. doi: 10.12701/yujm.2019.00255 PMC6784656 31620632

[28] Sun L Diao X Gang X Lv Y Zhao X Yang S. Risk factors for cognitive impairment in patients with type 2 diabetes. J Diabetes Res. (2020) 2020:4591938. doi: 10.1155/2020/4591938 32377520 PMC7196145

[29] Sun J Xia W Cai R Wang P Huang R Sun H. Serum insulin degrading enzyme level and other factors in type 2 diabetic patients with mild cognitive impairment. Curr Alzheimer Res. (2016) 13:1337–45. doi: 10.2174/1567205013666160615091043 27306699

[30] Darwish H Farran N Assaad S Chaaya M. Cognitive reserve factors in a developing country: education and occupational attainment lower the risk of dementia in a sample of Lebanese older adults. Front Aging Neurosci. (2018) 10:277. doi: 10.3389/fnagi.2018.00277 30279655 PMC6153348

[31] Lorenzo-López L Millán-Calenti JC López-López R Diego-Diez C Laffon B Pásaro E. Effects of degree of urbanization and lifetime longest-held occupation on cognitive impairment prevalence in an older Spanish population. Front Psychol. (2017) 8:162. doi: 10.3389/fpsyg.2017.00162 28243214 PMC5303752

[32] Matyas N Keser Aschenberger F Wagner G Teufer B Auer S Gisinger C. Continuing education for the prevention of mild cognitive impairment and Alzheimer's-type dementia: a systematic review and overview of systematic reviews. BMJ Open. (2019) 9:e027719. doi: 10.1136/bmjopen-2018-027719 PMC6609120 31270114

[33] Logroscino G Kang JH Grodstein F. Prospective study of type 2 diabetes and cognitive decline in women aged 70-81 years. BMJ. (2004) 328:548. doi: 10.1136/bmj.37977.495729.EE 14980984 PMC381043

[34] Barbiellini Amidei C Fayosse A Dumurgier J MaChado-Fragua MD Tabak AG van Sloten T. Association between age at diabetes onset and subsequent risk of dementia. Jama. (2021) 325:1640–9. doi: 10.1001/jama.2021.4001 PMC8080220 33904867

[35] Trento M Charrier L Salassa M Merlo S Passera P Cavallo F. Depression, anxiety and cognitive function in patients with type 2 diabetes: an 8-year prospective observational study. Acta diabetologica. (2015) 52:1157–66. doi: 10.1007/s00592-015-0806-0 26374233

[36] Trento M Trevisan M Raballo M Passera P Charrier L Cavallo F. Depression, anxiety, cognitive impairment and their association with clinical and demographic variables in people with type 2 diabetes: a 4-year prospective study. J endocrinological Invest. (2014) 37:79–85. doi: 10.1007/s40618-013-0028-7 24464454

[37] Van der Mussele S Fransen E Struyfs H Luyckx J Mariën P Saerens J. Depression in mild cognitive impairment is associated with progression to Alzheimer's disease: a longitudinal study. J Alzheimer's Dis JAD. (2014) 42:1239–50. doi: 10.3233/jad-140405 25024328

[38] Yang Q Zhou L Liu C Liu D Zhang Y Li C. Brain iron deposition in type 2 diabetes mellitus with and without mild cognitive impairment-an in *vivo* susceptibility mapping study. Brain Imaging Behav. (2018) 12:1479–87. doi: 10.1007/s11682-017-9815-7 29297155

[39] Yasuno F Kazui H Morita N Kajimoto K Ihara M Taguchi A. High amyloid-β deposition related to depressive symptoms in older individuals with normal cognition: a pilot study. Int J geriatric Psychiatry. (2016) 31:920–8. doi: 10.1002/gps.4409 26766490

[40] Bellary S Kyrou I Brown JE Bailey CJ. Type 2 diabetes mellitus in older adults: clinical considerations and management. Nat Rev Endocrinol. (2021) 17:534–48. doi: 10.1038/s41574-021-00512-2 34172940

[41] Galindo RJ Beck RW Scioscia MF Umpierrez GE Tuttle KR. Glycemic monitoring and management in advanced chronic kidney disease. Endocrine Rev. (2020) 41:756–74. doi: 10.1210/endrev/bnaa017 PMC7366347 32455432

[42] Nyenwe EA Ashby S Tidwell J. Diabetes consultation versus diabetes education in patients with poor glycaemic control: A telemedicine intervention study. J telemedicine telecare. (2022) 28:687–93. doi: 10.1177/1357633x20959213 32990153

[43] Koekkoek PS Kappelle LJ van den Berg E Rutten GE Biessels GJ. Cognitive function in patients with diabetes mellitus: guidance for daily care. Lancet Neurol. (2015) 14:329–40. doi: 10.1016/s1474-4422(14)70249-2 25728442

[44] Muresan M Micle O Antal L Dobjanschi L Dorofteiu M. Correlation between reactive oxygen species and homocysteine levels in normal pregnancy. Farmacia. (2011) 59:179.

[45] Garcia-Pinillos F Cozar-Barba M Munoz-Jimenez M Soto-Hermoso V Latorre-Roman P. Gait speed in older people: an easy test for detecting cognitive impairment, functional independence, and health state. Psychogeriatrics Off J Japanese Psychogeriatric Soc. (2016) 16:165–71. doi: 10.1111/psyg.12133 26114989

[46] McGough EL Cochrane BB Pike KC Logsdon RG McCurry SM Teri L. Dimensions of physical frailty and cognitive function in older adults with amnestic mild cognitive impairment. Ann Phys Rehabil Med. (2013) 56:329–41. doi: 10.1016/j.rehab.2013.02.005 PMC5562508 23602402

[47] Tian Q An Y Resnick SM Studenski S. The relative temporal sequence of decline in mobility and cognition among initially unimpaired older adults: Results from the Baltimore Longitudinal Study of Aging. Age Ageing. (2017) 46:445–51. doi: 10.1093/ageing/afw185 PMC5860013 27744302

[48] Gale CR Allerhand M Sayer AA Cooper C Deary IJ. The dynamic relationship between cognitive function and walking speed: the English Longitudinal Study of Ageing. Age (Dordrecht Netherlands). (2014) 36:9682. doi: 10.1007/s11357-014-9682-8 24997019 PMC4119879

[49] Zhong Y Zhang XY Miao Y Zhu JH Yan H Wang BY. The relationship between glucose excursion and cognitive function in aged type 2 diabetes patients. Biomed Environ Sci BES. (2012) 25:1–7. doi: 10.3967/0895-3988.2012.01.001 22424620

[50] Crane PK Walker R Larson EB. Glucose levels and risk of dementia. New Engl J Med. (2013) 369:1863–4. doi: 10.1056/NEJMc1311765 24195563

[51] García-Hermoso A Ramírez-Vélez R Celis-Morales CA Olloquequi J Izquierdo M. Can physical activity attenuate the negative association between sitting time and cognitive function among older adults? A mediation analysis. Exp gerontology. (2018) 106:173–7. doi: 10.1016/j.exger.2018.03.002 29549034

[52] Fritschi C Park H Richardson A Park C Collins EG Mermelstein R. Association between daily time spent in sedentary behavior and duration of hyperglycemia in type 2 diabetes. Biol Res Nurs. (2016) 18:160–6. doi: 10.1177/1099800415600065 PMC4755926 26282912

